# Human papillomavirus vaccination related knowledge, and recommendations among healthcare providers in Southern China: a cross-sectional survey

**DOI:** 10.1186/s12905-022-01728-8

**Published:** 2022-05-14

**Authors:** Siqi Chen, Chaofan Mei, Weikang Huang, Peiyi Liu, He Wang, Wei Lin, Shixin Yuan, Yueyun Wang

**Affiliations:** 1 The Research Institute of Maternity and Child Medicine, Affiliated Shenzhen Maternity and Child Healthcare Hospital, Southern Medical University, No. 2004, Hongli Road, Futian District, Shenzhen, 518000 China; 2 Department of Healthcare, Affiliated Shenzhen Maternity and Child Healthcare Hospital, Southern Medical University, Shenzhen, China; 3 Research Team of Cervical Cancer Prevention Project in Shenzhen, Affiliated Shenzhen Maternity and Child Healthcare Hospital, Southern Medical University, Shenzhen, China

**Keywords:** Healthcare providers, Human papillomavirus vaccination, Knowledge, Recommendation

## Abstract

**Background:**

Little research has been conducted to explore variables associated with the healthcare providers’ (HCPs) understanding and recommendation of human papillomavirus vaccine (HPV) since the vaccine was approved for use in China.

**Methods:**

A large-scale cross-sectional survey was conducted in southern China covering Guangdong, Guangxi, and Hainan provinces between April 2019 and October 2019. Firstly, descriptive analysis was used to access awareness, knowledge, barriers, and recommendations toward HPV vaccine among all participants. Multi-variable logistic regression was further applied to explore potential factors associated with awareness, acknowledgment of HPV vaccine, and recommendation behaviors toward HPV vaccine.

**Results:**

2075 questionnaires were collected, and 2054 were included in the final analysis. In total, 77.9% of participants have heard of HPV vaccine and obtained sub-optimal HPV/HPV vaccine knowledge scores with a mean (SD) of 13.8 (3.5) out of a maximum score of 23. 68.1% HCPs reported that they have recommended HPV vaccine to others. Province and profession were the most significant characteristics associated with awareness, knowledge score, and recommendation behavior toward HPV vaccine. HCPs in Guangdong obtained a much better knowledge score [Mean (SD) = 15.5 (3.0)] and reported higher recommendation behavior (82.8%). Compare with HCPs from the Division of Expanded Program on Immunization (DEPI), Community Health Center (CHC), and obstetrician-gynecologists, other non-HPV closely related professions showed a less competent knowledge of HPV and HPV vaccine [Mean (SD) = 12.5 (3.0)] and lower frequency of recommendation behavior on vaccination (58.1%). The difference in HPV vaccine knowledge among different professions was concentrating on the items about clinical pathology of HPV and the practical aspects of HPV vaccine. Educational level and title were also closely associated with their knowledge of HPV and its vaccine. Besides, knowledge scores independently determined with recommendation behavior (*OR* = 1.18, 95% *CI* 1.13–1.23).

**Conclusion:**

Knowledge level of HPV and HPV vaccine as well as recommendation behavior toward HPV vaccine were relatively lower in southern China and differed significantly between provinces. Profession-specific gaps on the knowledge level of HPV and HPV vaccine emphasized the need for targeted education and training to improve HCPs’ engagement in the promotion of the HPV vaccine.

**Supplementary Information:**

The online version contains supplementary material available at 10.1186/s12905-022-01728-8.

## Background

Cervical cancer is the fourth most common gynecological cancer and causes a tremendous disease burden in China [1, 2]. China contributed to about 18.7% of the global burden of cervical cancer, with about 106 000 new cases of cervical cancer occurring in 2018 [3]. Besides, the incidence and death of cervical cancer have increased more than three times from 2005 to 2015 [4]. Human papillomavirus (HPV) is one of the most common sexually transmitted infections worldwide, with high-risk types closely related to cervical cancer, other anogenital cancers (i.e., penile, vaginal, vulva, and anal cancers), and head and neck cancers (i.e., cancers of the oral cavity, oropharynx, and larynx) as well as low-risk types responsible for benign warts [5]. Almost all cervical cancer was caused by high-risk HPV [3].

HPV vaccine has been proved efficacy for protecting against HPV-related diseases and decreasing the corresponding disease burden [6–8]. After a decade’s delay, since U.S. FDA licensure in 2006, the first HPV vaccine was approved in China in 2016. The vaccination rate of Chinese was relatively low since HPV vaccine was introduced into China late and not included in National Immunization Program (NIP) [9–11]. Reported knowledge and attitude related to HPV vaccine are also far from optimistic [12–14]. A recent national school-based survey in China showed that only 17.1% of adolescents reported having heard of HPV vaccine [13].

Healthcare providers (HCPs) play a significant role in HPV vaccine uptake because their recommendations, reminders, or/and counseling for the vaccine are associated with attitude toward vaccine among the general population and parental intention to vaccinate their children [15–18]. Furthermore, one of the most significant factors influencing HCPs’ recommendation is belief and understanding of HPV and HPV vaccine [19]. Thus, it is worthwhile to investigate the HCPs’ awareness, knowledge, and recommendation toward HPV vaccine and provide related evidence for future vaccine roll-out activities. To date, the existing data about belief and knowledge of HPV vaccine for HCPs in Southern China are still scant. Studies exploring potential factors associated with HCPs’ knowledge, attitudes, and recommendation behaviors were more limit [20, 21].

Most studies investing HPCs’ knowledge and attitude toward HPV vaccine targeted to whole HPCs or single profession but few stratified by different professions. In current healthcare practice in China, Community Health Center (CHC) is the main unit for primary health care including implementing the vaccination service. The Division of Expanded Program on Immunization (DEPI) affiliated to Chinese Center for Disease Control and Prevention (CDC) takes the primary responsibility for leading immunization activities including technical guideline formulation, surveillance as well as health education and public communication of vaccination. Obstetrician-gynecologist in hospital provided therapeutic services for relative HPV-related diseases. Thus, HCPs from the DEPI affiliated to CDC, HCPs from CHC, and obstetrician-gynecologist in hospitals are three major professions directly responsible for public HPV vaccine recommendation or inoculation. Other non-HPV closely related HCPs also need to discuss HPV and HPV vaccine queries during patients’ clinic visits. As HPV vaccine obtained more attention in recent years, the discrepancy of knowledge and recommendation toward HPV vaccine between different professions need to be analyzed for further targeted education to overcome vaccine confusion and hesitancy among HCPs and audiences.

For bridging the knowledge gap mentioned above, the present study aimed to assess awareness, knowledge of HPV and HPV vaccine as well as recommendation behavior toward vaccination among HCPs with different professions in southern China. Furthermore, other associated potential factors like demographic characteristics were also investigated and analyzed.

## Methods

### Study design and participants

A cross-sectional survey of awareness, knowledge, and recommendation toward HPV vaccination for HCPs from Southern China (including Guangdong, Guangxi, and Hainan province) was administered between April 2019 and October 2019. Randomized cluster sampling was used to select 17 cities/counties from 39 cities/counties. A target sample size of 2000 was predetermined based on the ability to estimate an HPV awareness rate of 50% ± 1.5% with 95% precision.

### Data collection and questionnaire

Data were collected and managed using Research Electronic Data Capture (REDCap), an electronic data capture tool hosted at Cancer Hospital, Chinese Academy of Medical Sciences & Peking Union Medical College [22, 23]. REDCap is a secure, web-based software platform designed to support data capture for research studies. Through scanning the Quick Response (QR) code generated through REDCap, respondents were guided to finish the electronic questionnaire. Before accessing the questionnaire, they were required to provide informed consent by clicking on the agreement button after reviewing the informed consent form (ICF). Questionnaires filled out were directly sent to the backstage data center of REDCap.

The questionnaire was developed by a research team consisting of epidemiologists, HCPs from DEPI, and gynecologists based on information from previous studies conducted by the research team [24]. The draft questionnaire was then tested on two HCPs, one obstetrician–gynecologist and one HCP from DEPI. They were first asked to complete the questionnaire individually. After finishing the questionnaire, they were interviewed on their understanding of each question and any difficulties met when completing the questionnaire. Questionnaires were finalized after adjusting the contents and wording based on the feedback from these interviewees [24]. The questionnaire consists of 55 questions, including 4 domains: (i) Socio-demographic information, (ii) Awareness of HPV and HPV vaccine, (iii) Knowledge of HPV and HPV vaccine, (iv) Willingness and behavior of recommending HPV vaccination. The socio-demographic information included age, education level, occupation, household monthly income, and family information. The part of awareness of HPV and HPV vaccine included questions of if heard of HPV and HPV vaccine. In the knowledge section, respondents were asked a series of questions about HPV and HPV vaccine including diseases attributable to HPV, HPV transmission routes, risk factors of HPV infection, classification of high-risk and low-risk types, the meaning of “valent”, the necessity of cervical screening after vaccination, the best time to get vaccinated and function of HPV vaccine. Knowledge score was calculated by summing the score of correct responses to these questions. One point was assigned to every single-option question and three points were assigned to every multiple-option question. For multiple-option questions, the score assigned to each correct option was three divided by the number of correct options in this question. The maximum knowledge score of HPV and HPV vaccine was 23. The last part of the survey included questions of if willing to recommend HPV vaccination and if recommend HPV vaccination to others.

### Statistical analysis

The socio-demographic information was summarized by descriptive statistics. HCPs were divided into four groups according to professions: HCPs from DEPI, HCPs from CHC, obstetrician-gynecologist, and other HCPs. HCPs from DEPI, HCPs from CHC and obstetrician-gynecologist were HCPs work closely with HPV vaccination. Other HCPs included healthcare providers from non-immunization division of CDC, non-obstetrician-gynecologist clinicians, nurses, and researchers in hospitals. Awareness of HPV vaccine, knowledge score of HPV and HPV vaccine, HPV vaccination recommendation behavior were presented with frequency and percentage. The difference in awareness and recommendation of HPV vaccination between subgroups were compared using Chi-square tests while the difference in knowledge score was compared using *t*-test or analysis of variance (ANOVA). Multi-variable logistic regression analyzed factors (including province, age group, sex, ethnicity, education level, profession, and title) associated with: (1) Awareness of HPV vaccine; (2) knowledge score of HPV and HPV vaccination among respondents who have heard of HPV vaccine; (3) HPV vaccination recommendation behavior (yes; no) among respondents who have heard of HPV vaccine. Odds ratios (*OR*), 95% confidence intervals (95% *CI*), and *P*-values were calculated for each independent variable. Twotailed tests with *P* values less than 0.05 were thought to be statistically significant. Analyses were performed using R software, version 3.5.2.

## Results

### Characteristics of participants

In total, 2074 questionnaires were collected, and 20 were excluded after checking for missing information and logistic errors. A total of 2054 records were included in the final analysis. A summary of the socio-demographic characteristics of the respondents was provided in Table 1. The participants’ age ranged from 21 to 69 years, with a mean of 37.0 ± 8.9 years old. Majority of respondents were not more than 40 years old (65.7%, n = 1349), female (90.6%, n = 1861), Han (63.2%, n = 1299). 44% participants worked closely with HPV and HPV vaccine (n = 904).

**Table 1:** Socio-demographic characteristics (N = 2054)

Characteristics	Respondents (n)	Respondents (%)
*Province*		
Guangdong	643	31.3
Guangxi	1177	57.3
Hainan	234	11.4
*Age*		
< 40	1350	65.7
≥ 40	704	34.3
*Sex*		
Male	193	9.3
Female	1861	90.6
*Ethnicity*		
Han	1299	63.2
Minority	755	36.8
*Educational level*		
High school and below	81	3.9
Professional degree	762	37.1
Bachelor	1119	54.5
Master and above	92	4.4
*Profession*^a^		
HCPs from DEPI	84	4.1
HCPs from CHC	87	4.2
Obstetrician–gynecologist	733	35.7
Other HCPs	1149	56.0
*Title*		
Junior	1127	54.9
Middle	623	30.3
Associate senior/senior	304	14.8

^a^One missing value in HCPs occupation

### Awareness of HPV vaccination

Most participants reported that they had heard of HPV vaccine (77.9%, n = 1601), but awareness differed by province, age, ethnicity, education level, profession, and title (Table 2). Compared with HPCs from DEPI, other HCPs, (*OR* = 0.35, 95% *CI* 0.11–0.87) showed significantly lower awareness of HPV vaccine. Other variables significantly associated with awareness of HPV vaccine were province (Guangxi: *OR* = 0.20, 95% *CI* 0.13–0.29; Hainan: *OR* = 0.50, 95% *CI* 0.29–0.86), the minority (*OR* = 0.77, 95% *CI* 0.60–1.00), educational level (Professional degree: *OR* = 2.40, 95% *CI* 1.33–4.22; Bachelor: *OR* = 3.47, 95% *CI* 1.91–6.20; Master and above: *OR* = 24.33, 95% *CI* 6.27–162.23) and title (Middle title: *OR* = 1.52, 95% *CI* 1.13–2.06; Associate senior/Senior: *OR* = 1.93, 95% *CI* 1.11–3.45) in multi-variate logistic regression.

**Table 2:** Factor associated with awareness of HPV vaccine (n = 2054)

Characteristics	Frequency (%)	Uni-variate analysis		Multi-variate logistic regression	
		*χ*^2^	*P*	*OR* (95% *CI*)	*P*
Overall	1601 (77.9)	–	–		
*Province*		185.5	< 0.001		
Guangdong	606 (94.2)			1 (ref)	–
Guangxi	793 (67.4)			0.20 (0.13–0.29)	< 0.001
Hainan	202 (86.3)			0.50 (0.29–0.86)	0.012
*Age*		56.04	< 0.001		
< 40	985 (73.0)			1 (ref)	–
≥ 40	616 (87.5)			1.40 (0.99–2.00)	0.060
*Sex*		2.16	0.141		
Male	159 (82.4)			1 (ref)	–
Female	1442 (77.5)			1.55 (0.98–2.41)	0.058
*Ethnicity*		88.00	< 0.001		
Han	1098 (84.5)			1 (ref)	–
Minority	503 (66.6)			0.77 (0.60–1.00)	0.041
*Educational level*		35.88	< 0.001		
High school and below	57 (70.4)			1 (ref)	–
Professional degree	559 (73.4)			2.40 (1.33–4.22)	0.003
Bachelor	895 (80.0)			3.47 (1.91–6.20)	< 0.001
Master and above	90 (97.8)			24.33 (6.27–162.23)	< 0.001
*Profession*		113.90	< 0.001		
HCPs from DEPI	79 (94.0)			1 (ref)	–
HCPs from CHC	84 (96.6)			2.67 (0.59–14.09)	0.209
Obstetrician–gynecologist	639 (87.2)			0.58 (0.19–1.47)	0.290
Other HCPs	799 (69.5)			0.35 (0.11–0.87)	0.038
*Title*		72.13	< 0.001		
Junior	809 (71.8)			1 (ref)	–
Middle	513 (82.3)			1.52 (1.13–2.06)	0.006
Associate senior/senior	279 (91.8)			1.93 (1.11–3.45)	0.023

### Knowledge about HPV and HPV vaccine

In total, the mean (SD) of HPV and HPV vaccine knowledge among the participants who have heard of HPV vaccine was 13.78 (3.45), with a mean (SD) of HPV knowledge was 6.61 (2.15) out of a maximum score of 14 and mean (SD) of HPV vaccine knowledge was 7.14 (1.98) out of a maximum score of 9. The proportion of correct responses to knowledge items were shown in Additional file 1: Fig S1.

Compared with other subgroups, Guangdong, the older (≥ 40), male, Han, HCPs from DEPI, master and above and Associate senior/Senior group obtained a higher knowledge score of HPV and HPV vaccine. The result of multivariate linear regression showed that variables associated with HPV and HPV vaccine knowledge score were province (Guangxi: *OR* = 0.12, 95% *CI* 0.08–0.18; Hainan: *OR* = 0.28, 95% *CI* 0.16–0.48), master and higher education level (*OR* = 8.12, 95% *CI* 2.78–23.74), other HCPs (*OR* = 0.19, 95% *CI* 0.09–0.40) and Associate senior/Senior (*OR* = 3.73, 95% *CI* 2.09–6.66) (Table 3).

**Table 3:** Factor associated with knowledge score of HPV and HPV vaccine (n = 1601)

Characteristics	Mean (SD)	Uni-variate analysis		Multi-variate logistic regression	
		*t*/*F*	*P*	*OR* (95% *CI*)	*P*
Overall	13.78 (3.45)				
*Province*		159.233	< 0.001		
Guangdong	15.46 (2.96)			1 (ref)	–
Guangxi	12.41 (3.19)			0.12 (0.08–0.18)	< 0.001
Hainan	14.05 (3.53)			0.28 (0.16–0.48)	< 0.001
*Age*		6.361	< 0.001		
< 40	13.33 (3.15)			1 (ref)	–
≥ 40	14.50 (3.77)			0.92 (0.61–1.39)	0.695
*Sex*		2.256	0.025		
Male	14.31 (3.07)			1 (ref)	–
Female	13.72 (3.49)			1.31 (0.78–2.21)	0.312
*Ethnicity*		10.301	< 0.001		
Han	14.36 (3.38)			1 (ref)	–
Minority	12.50 (3.26)			0.92 (0.62–1.37)	0.693
*Educational level*		52.952	< 0.001		
High school and below	13.60 (3.12)			1 (ref)	–
Professional degree	13.02 (3.34)			1.06 (0.46–2.44)	0.898
Bachelor	14.04 (3.46)			2.28 (0.98–5.32)	0.056
Master and above	15.98 (2.95)			8.12 (2.78–23.74)	< 0.001
*Profession*		182.934	< 0.001		
HCPs from DEPI	15.90 (2.28)			1 (ref)	–
HCPs from CHC	14.77 (2.61)			0.98 (0.38–2.52)	0.968
Obstetrician–gynecologist	14.94 (3.59)			0.99 (0.47–2.09)	0.986
Other HCPs	12.52 (3.02)			0.19 (0.09–0.40)	< 0.001
*Title*		90.8	< 0.001		
Junior	13.20 (3.09)			1 (ref)	–
Middle	13.69 (3.51)			1.43 (0.97–2.10)	0.071
Associate senior/senior	15.59 (3.70)			3.73 (2.09–6.66)	< 0.001

### HPV vaccination recommendation willingness and behavior

A majority of participants (94.8%, n = 1517) were willing to recommend HPV vaccine to others The most important reason why respondents were unwilling to recommend HPV vaccine to others was that they worry about the safety of HPV vaccine (Additional file 1: Fig S2).

Over half of respondents (68.1%, n = 1091) reported that they have recommended HPV vaccine to others, and 91.1% HCPs from DEPI have recommended. Factors associated with HPV vaccination recommendation behavior included province (Guangxi: *OR* = 0.57, 95% *CI* 0.40–0.80; Hainan: *OR* = 0.59, 95% *CI* 0.39–0.91), old age (≥ 40, *OR* = 1.47, 95% *CI* 1.06–2.05), other HPCs (*OR* = 0.34, 95% *CI* 0.14–0.74), knowledge score of HPV and HPV vaccine (*OR* = 1.18, 95% *CI* 1.13–1.23) (Table 4).

**Table 4:** Factors associated with HPV vaccination recommendation behavior (n = 1601)

Characteristics	Frequency (%)	Uni-variate analysis		Multi-variate logistic regression	
		*χ*^2^	*P*	*OR* (95% *CI*)	*P*
Overall	1091 (68.1)			–	–
*Province*		105.67	< 0.001		
Guangdong	502 (82.8)			1 (ref)	–
Guangxi	452 (57.0)			0.57 (0.40–0.80)	0.001
Hainan	137 (67.8)			0.59 (0.39–0.91)	0.015
*Age*		15.51	< 0.001		
< 40	635 (64.5)			1 (ref)	–
≥ 40	456 (74.0)			1.47 (1.06–2.05)	0.021
*Sex*		4.00	0.046		
Male	120 (75.5)			1 (ref)	–
Female	971 (67.3)			0.95 (0.60–1.46)	0.803
*Ethnicity*		37.29	< 0.001		
Han	802 (73.0)			1 (ref)	–
Minority	289 (57.5)			0.97 (0.73–1.29)	0.846
*Educational level*		16.67	< 0.001		
High school and below	40 (70.2)			1 (ref)	–
Professional degree	356 (63.7)			0.10 (0.51–1.88)	0.991
Bachelor	619 (69.2)			1.13 (0.57–2.17)	0.712
Master and above	76 (84.4)			2.12 (0.84–5.46)	0.114
*Profession*		84.83	< 0.001		
HCPs from DEPI	72 (91.1)			1 (ref)	–
HCPs from CHC	72 (85.7)			0.89 (0.30–2.48)	0.824
Obstetrician–gynecologist	483 (75.6)			0.50 (0.20–1.09)	0.106
Other HCPs	464 (58.1)			0.34 (0.14–0.74)	0.011
*Title*		14.44	< 0.001		
Junior	535 (66.1)			1 (ref)	–
Middle	339 (66.1)			0.77 (0.57–1.03)	0.079
Associate senior/senior	217 (77.8)			0.73 (0.45–1.18)	0.194
*HPV and HPV vaccine knowledge score*	–	–	–	1.18 (1.13–1.23)	< 0.001

## Discussion

In this study, we analyzed the awareness, knowledge, and recommendation behavior toward HPV vaccine among HCPs by conducting a large-scale survey covering three provinces (Guangdong, Guangxi, and Hainan) in southern China. In general, awareness, knowledge level, and recommendation behavior were relatively lower in southern China and differed significantly among the three provinces. Besides, the present study also found that profession was a key predictor of HCPs’ awareness, knowledge, and recommendation behavior toward HPV vaccine.

In the present study, around 77.9% of HCPs had heard of HPV vaccine in southern China, lower than the rate in mainland China according to the previous study (84%) [24]. Among the participants who have heard of HPV vaccine obtained a knowledge score of HPV and HPV vaccine with a mean (SD) was 13.78 (3.45) out of a maximum score of 23. The knowledge level of HCPs in southern China was much lower than in some other developed countries such as England and New Zealand [20, 21], which may be due to the late approval and no NIP introduction for HPV vaccines.

The knowledge score of HPV vaccine items was quite high while the score of HPV items was relatively low. According to the correct rate of each knowledge item, more than 90% HCPs have acknowledged that HPV effect can cause cervical cancer, HPV vaccine can prevent cervical cancer and cervical screening is needed after HPV vaccination, but most HCPs have less understanding of HPV, especially classification of HPV types. The result indicated that most HCPs only simply understood the HPV and were aware of the benefits of HPV vaccine but had no depth-understanding of the clinical pathology of HPV. Research conducted in countries with HPV vaccination programs also revealed that knowledge about HPV and the HPV vaccination among health professionals was frequently incomplete, and the lack of complete knowledge had the potential to spread misinformation and cause confusion among patients [21, 25, 26].

Compare with other HCPs, the knowledge scores of HPV and HPV vaccine were significantly higher in HCPs from DEPI and CHC and obstetrician-gynecologist. Though other HCPs were not the dominant role in introducing or recommending HPV vaccine, they would run into some consultation for HPV vaccine during the clinic. Due to the late approval and unavailability in the NIP, the current HPV vaccine uptake in China is far from optimistic. A nationwide survey showed that the HPV vaccine uptake rate of females aged 18–45 was only 3% [27]. Furthermore, women aged 20–30 years were the target population encouraged to obtain the vaccine. World Health Organization (WHO) recommended that, by 2030, 90% of girls should be fully vaccinated before the age of 15 in 2019 [28]. Since then, it is also encouraged by the National Health Commission to increase public accessibility and to promote HPV vaccination for target populations in China.15 pilot cities in China have already initiated the project of free HPV vaccine inoculation for 9–15 years female high school students to promote cervical cancer elimination. Therefore, more consulting about HPV and HPV vaccine would be faced by other HCPs, especially pediatricians. Thus, it raised higher standards for other HCPs to be equipped with associated knowledge.

The difference in HPV and HPV vaccine knowledge among different professions was concentrating on the items about clinical pathology of HPV and the practical aspects of HPV vaccine such as risk factors of HPV infection, classification of high and low-risk types, the meaning of valent, and the best time for HPV vaccine. It was not hard to explain that other HCPs had insufficient knowledge on both HPV and HPV vaccination. Compared with HCPs from CHC, obstetrician-gynecologist obtained a relatively high correct rate of HPV knowledge items but not for the HPV vaccine knowledge items. Previous studies also found that HCPs worked at general and specialist hospitals had a lower understanding of HPV vaccine while providers who worked at community health service centers achieved significantly higher knowledge scores for HPV vaccine than other HCPs, which might be attributable to the HPV vaccination policies in China [29]. Considering the distribution of information, it will be beneficial to increase the supply of information about HPV vaccination to HCPs in a hospital to strengthen the accessibility of vaccination information and strengthen HPV education to HCPs from CHC is of great necessity.

Regarding the recommendation practice toward HPV vaccine, it was not surprising that other HCPs reported lower recommendation behavior compared with HCPs from DEPI, CHC and obstetrician-gynecologist. In China, HCPs from DEPI and CHC work directly related to HPV and HPV vaccine for DEPI affiliated to CDC takes the primary responsibility for leading immunization activities while CHC is the main unit for implementing the vaccination service. Obstetrician–gynecologist have more opportunities to make HPV vaccination recommendations due to clinic consulting. By contrast, other HCPs have fewer opportunities to recommend HPV vaccine. Another independent variable associated with recommendation behavior was knowledge of HPV and HPV vaccine. Higher knowledge could increase the confidence of HCPs in presenting and discussing the HPV vaccine [30]. We also investigated HCPs’ willingness of recommendation, only 5.2% of participants indicated that they were unwilling to recommend HPV vaccine to others, and the most reason they reported was that they worry about the safety of HPV vaccine. In fact, the worry about safety resulted from the insufficient understanding of the recent process of HPV vaccine. This finding further emphasized the need for more education to improve HCP’s knowledge for increasing recommendation and vaccine uptakes.

The HPCs’ awareness differed highly between provinces, with 94.2% HPCs having heard of HPV vaccine in Guangdong but only 67.4% in Guangxi and 86.3% in Hainan. In terms of the knowledge score of HPV and HPV vaccine, far more HCPs from Guangdong province achieved higher knowledge scores compared with Guangxi province and Hainan province. One possible reason for this discrepancy was that awareness and understanding toward HPV vaccine might be associated with medical resources and the level of medical treatment of each province. It is acknowledged that the medical level in Guangdong is superior to the other two provinces, and could be certified according to China’s hospital rankings [31]. Besides, it may likely be attributable to the geographical location of Guangdong. Guangdong is located the closest to Hong Kong, where the HPV vaccine was introduced in 2004 and has been covered by mass media since then, thus citizens in Guangdong acquired relevant information through interpersonal communication and some public service and advertisements from Hong Kong. Last but not least, some public measures such as including HPV vaccination in citizens’ medical insurance were firstly presented in Shenzhen, Guangdong. Furthermore, the increasing number of people who get vaccinated could promote the HCPs’ willingness to learn about HPV and HPV vaccination.

In terms of education level and title, a higher education degree or higher title could predict HCPs’ awareness of HPV vaccine and a higher knowledge level of HPV and HPV vaccine. Considering over half of HCPs in Southern China have a degree lower than master or have a title lower than associate senior, organizing regular internal training sessions and developing education packages about HPV are necessary to facilitate HCPs with accurate knowledge and recent progress. More HCPs with high education levels and high titles reported that they have recommended HPV vaccine to others, but education degree and title were not the independent variables for recommendation behavior mostly contributed to HCPs’ profession.

There were several limitations in our study. Firstly, the present study was cross-sectional and the data collection was based on self-reported data, which might be subject to self-reporting bias and a tendency to report socially desirable responses. A second limitation was that participant in the survey was purely voluntary, it was hard to avoid nonresponse bias. The majority of participants in the present study were females, which may be due to females having more attention to HPV vaccine than males. Though the gentle distribution in the present study may overestimate the result, it reminded us that the importance of targeted education to provide adequate information HCPs. Third, the questionnaire was developed from previous studies [32, 33]. However, since most of the survey questions had been used several times before in China, modified according to recent vaccine updates by multidisciplinary experts, and pre-tested, the reliability of this study should be acceptable.

## Conclusion

In this large-scale survey, we concluded that awareness, knowledge level, and recommendation behavior were relatively lower in southern China and differed significantly between provinces. Profession-specific knowledge gaps on the understanding of HPV and HPV vaccines reflected some phenomenon or shortage of current situations of HPV and HPV vaccination in southern China, which provided evidence for further vaccine roll-out activities. As knowledge level of HPV and HPV vaccines is an important key associated with recommendation behavior, more targeted education is necessary to provide adequate information HCPs for increasing HPV vaccine uptake.

## Supplementary Information

**Additional file 1. Fig S1.** Correct responses to HPV and HPV vaccine knowledge items(N=1601). **Fig S2** Barriers forward willingness to recommend HPV vaccination.

## Data Availability

The datasets used and/or analysed during the current study are available from the corresponding author on reasonable request.
