# Supplementary material for: Human papillomavirus vaccination related knowledge, and recommendations among healthcare providers in Southern China: a cross-sectional survey
Source: BMC Womens Health. 2022 May 14;22:169. doi: 10.1186/s12905-022-01728-8 (PMC9107117; doi:10.1186/s12905-022-01728-8)
Supplement: Supplementary file 1 — Additional file 1. Fig S1. Correct responses to HPV and HPV vaccine knowledge items(N=1601). Fig S2 Barriers forward willingness to recommend HPV vaccination. [file 12905_2022_1728_MOESM1_ESM.docx]

**Supplement**


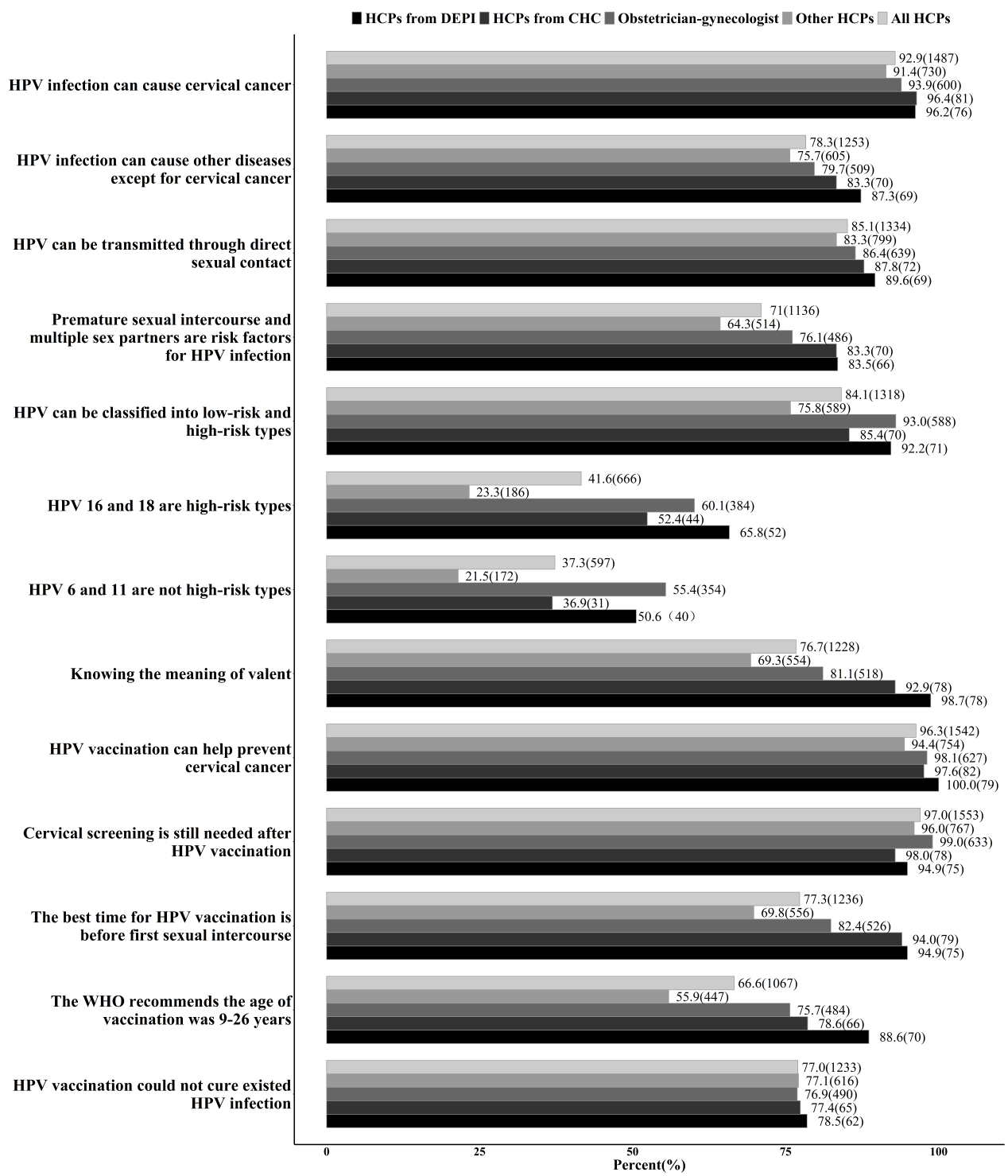


Fig S1 Correct responses to HPV and HPV vaccine knowledge items(N=1601)


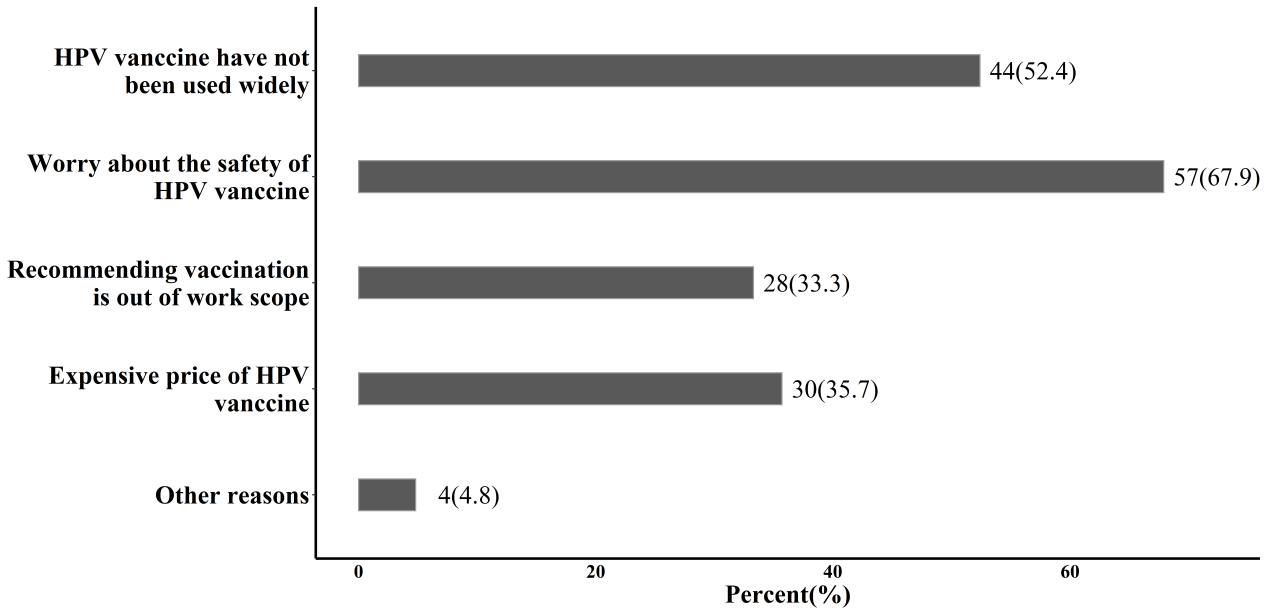


Fig S2 Barriers forward willingness to recommend HPV vaccination
